# The Pathology of the Eye

**Published:** 1910-09

**Authors:** 


**The Pathology of the Eye.** By J. HERBERT PARSONS, D.Sc., F.R.C.S. 4 Vols. London: Hodder and Stoughton. 1904–1909.

This work, in four large volumes, is profusely illustrated, and is so well arranged that, despite its size, any student can find readily within its pages an account of whatever pathological conditions of the tissues of the eye he desires to study. For the ophthalmic surgeon the book will be found a complete reference to any affection of the eye. The author writes upon each disease and on every variety of it in an easy style, and with a completeness that gives the reader pleasure and satisfaction. It is difficult to speak too highly of the value of the work in its entirety; it reflects the greatest credit on the author. It is a most valuable and almost essential addition to any public medical library, or to that of any ophthalmologist's (English or foreign), and it relieves the British school of ophthalmic surgeons from the criticism that good as they are in practical clinical work, little has been done by them, excepting in the case of a few well-recognised teachers, to advance the pathological knowledge of eye-diseases.

Parsons's book is perhaps the best on eye diseases and their pathology that has been written in any language. Two volumes are devoted to the histology of the various structures of the lids and eyeballs; two others to the general pathology of the various diseases that affect those structures. There is a quite exhaustive list of all the papers bearing on the subject that have been published in the European and American scientific journals. No subject in clinical ophthalmology is unnoticed; every branch receives due attention.

The illustrations are drawn from actual diseased conditions of the eyes and their appendages, or direct from microscopic slides of the pathological conditions alluded to.

The author's text and comments are practically enough to give a wide acquaintance with any and every diseased condition, but to render further investigation easy the most important papers under each heading are "starred," so that the monographs most deserving of attention can be readily distinguished from others, which, however, in their turn are of great value in helping to criticise or support the views which are more generally accepted.

The complete collection of material, its masterly arrangement, and the capable digest given by the author from his own knowledge, as a result of the study of the literature, entitle the book to a high rank among medical publications. Those who have done the most expert work in the various subjects will be the first to admit how fair and judicial the author's views are on the special subjects which may be under discussion, while the width of his scientific training and his exceptional opportunities for the microscopic examination of diseased eyelids give increased value to the opinions expressed by him.

Even those who are not primarily interested in eye diseases may with advantage look through the pages of these volumes; they could well be taken as a type upon which a compendium of the work done in other specialities might be based.

We much regret the delay in noticing this valuable work of Dr. Parsons, which contains as complete an account of diseases of the eye from the standpoint of pathology as is possible at the present time.

